# Direct observation of supramolecular binding of light hydrocarbons in vanadium(iii) and (iv) metal–organic framework materials†
†Electronic supplementary information (ESI) available: Synthetic details, experimental methods, details of NPD refinements, additional isotherms, INS spectra and views of guest binding sites is available in the online version of the paper. Correspondence and requests for materials should be addressed to S. Y. and M. S. See DOI: 10.1039/c8sc00330k


**DOI:** 10.1039/c8sc00330k

**Published:** 2018-02-21

**Authors:** Zhenzhong Lu, Harry G. W. Godfrey, Ivan da Silva, Yongqiang Cheng, Mathew Savage, Pascal Manuel, Svemir Rudić, Anibal J. Ramirez-Cuesta, Sihai Yang, Martin Schröder

**Affiliations:** a School of Chemistry , University of Manchester , Oxford Road , Manchester , M13 9PL , UK . Email: Sihai.Yang@manchester.ac.uk ; Email: M.Schroder@manchester.ac.uk; b Institute of Advanced Materials (IAM) , Nanjing Tech University , Nanjing , 210009 , P. R. China; c ISIS Facility , STFC Rutherford Appleton Laboratory , Chilton , Oxfordshire OX11 0QX , UK; d The Chemical and Engineering Materials Division (CEMD) , Neutron Sciences Directorate , Oak Ridge National Laboratory , Oak Ridge , TN 37831 , USA

## Abstract

Fine tuning of host–guest supramolecular interactions in porous systems enables direct control over the properties of functional materials. We report here a modification of hydrogen bonding and its effect on guest binding in a pair of redox-active metal–organic frameworks (MOFs). Oxidation of MFM-300(V^III^) {[VIII2(OH)_2_(L)], LH_4_ = biphenyl-3,3′,5,5′-tetracarboxylic acid} is accompanied by deprotonation of the bridging hydroxyl groups to afford isostructural MFM-300(V^IV^), [VIV2O_2_(L)]. The precise role of the hydroxyl groups, *O*-carboxylate centres and π–π interactions in the supramolecular binding of C_2_ hydrocarbons in these materials has been determined using neutron diffraction and inelastic neutron scattering, coupled with DFT modelling. The hydroxyl protons are observed to bind to adsorbed unsaturated hydrocarbons preferentially in MFM-300(V^III^), particularly to C_2_H_2_, which is in a sharp contrast to MFM-300(V^IV^) where interactions with *O*-carboxylate centres and π–π interactions predominate. This variation in structure and redox leads to notably higher separation selectivity for C_2_H_2_/CH_4_ and C_2_H_4_/CH_4_ in MFM-300(V^III^) than in MFM-300(V^IV^). Significantly, owing to the specific host–guest interactions, MFM-300(V^III^) shows a record packing density for adsorbed C_2_H_2_ at 303 K and 1 bar, demonstrating its potential for use in portable acetylene stores.

## Abstract 2

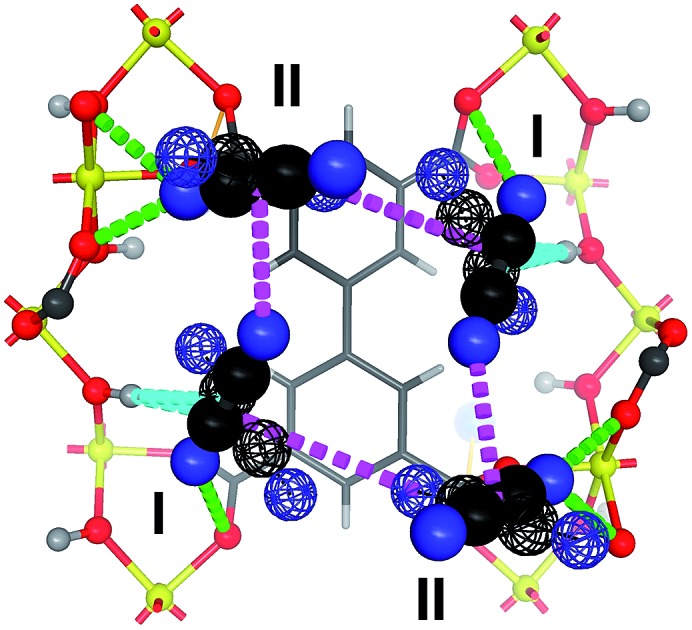
Binding of C_2_H_2_ in MFM-300(V^III^) showing interactions to O–H, carboxylate O-centres and intermolecular packing.

## Introduction

Porous materials have shown great promise for substrate binding, storage, separation, and delivery due to their unique host–guest interactions.^1^,^2^ Understanding the mechanisms of host–guest binding in porous materials at a molecular level is of critical importance for the development of new materials with desired properties. However, experimental investigations to gain such details are often not feasible in large void materials because of the lack of order.^3^ Moreover, the nature of these host–guest interactions is often based upon weak and dynamic supramolecular contacts such as hydrogen bonding, π···π stacking, and van der Waals forces, leading to the presence of severe guest disorder in the pores of the host.

Metal–organic frameworks (MOFs) with extraordinary tuneability of the pore geometry and surface functionality can facilitate control over adsorptive separation of small molecule hydrocarbons, and their high surface areas generally give rise to a large working capacity for dynamic separation.^4^,^5^ The development of MOFs showing enhanced substrate selectivity provides a strong motivation for studying the binding behavior of adsorbed hydrocarbons within pores. Vacant metal sites within MOFs can be employed to bind ethylene preferentially over ethane or propylene over propane *via* binding of the unsaturated species to metal centres.^6^,^7^ However, these sites generally lose activity rapidly as the vacant sites are bound by hydrocarbon molecules. In contrast, it has been confirmed in exceptional cases that soft functional groups within MOFs are also capable of preferentially binding unsaturated C_2_ hydrocarbons, in particular for distinguishing between alkenes and alkynes.^8^,^9^

We were interested in assessing the role of hydroxyl groups within porous MOFs for the selective binding of light hydrocarbons for potential adsorptive separation. We sought to modify the hydroxyl groups within the pore while still retaining the overall structure and porosity of resultant MOF. MFM-300(V^III^), {[VIII2(OH)_2_(L)] LH_4_ = biphenyl-3,3′,5,5′-tetracarboxylic acid}, and its oxidised iso-structural counterpart MFM-300(V^IV^), [VIV2O_2_(L)], provide a unique platform to study the precise role of the –OH group in the supramolecular binding of hydrocarbon molecules.^10^ Here we report a comprehensive investigation of the binding of light hydrocarbons within these two MOFs *via* neutron powder diffraction (NPD) and inelastic neutron scattering (INS), coupled with DFT modelling. These complimentary studies find that the hydroxyl protons play an important role in binding of C_2_H_2_/C_2_H_4_ molecules in the pores in contrast to the case of MFM-300(V^IV^) where adsorbed C_2_H_2_/C_2_H_4_ molecules are found to be remote from the bridging oxy groups. The pore environment of MFM-300(V^III^) is found to be optimal for binding of C_2_H_2_ and leads to an exceptional packing density of up to 0.38 g cm^–3^ of adsorbed C_2_H_2_ at 303 K and 1 bar.

## Results and discussion

### Analysis of crystal structure and porosity of MFM-300(V)

**Fig. 1:**
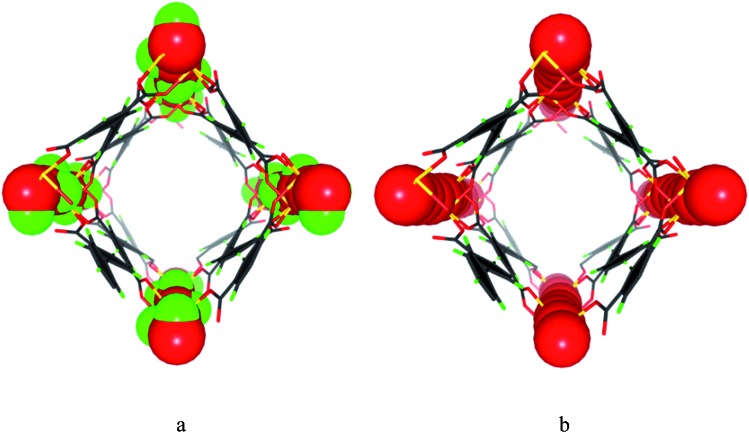
Perspective views of the channels in (a) MFM-300(V^III^) and (b) MFM-300(V^IV^). The hydroxyl groups (H atom, green; O atom, red) in MFM-300(V^III^) protrude into the channel, which change to O^2–^ bridge (red) in MFM-300(V^IV^).

MFM-300(V^III^) was synthesised following our previously reported method^10^ and crystallises in the space group *I*4_1_22. Adjacent pair of V^III^ centres are bridged by two carboxylates and a μ_2_-hydroxyl group forming an extended chain of [V_2_(OH)_2_O_4_]_∞_ along the *c* axis. The carboxylate ligands further bridge the [V_2_(OH)_2_O_4_]_∞_ chains to give a three dimensional network with square-shaped channels decorated with hydroxyl groups pointing into the pores (Fig. 1). MFM-300(V^IV^) was obtained by heating the as-synthesised MFM-300(V^III^) sample at 150 °C under a flow of O_2_ for 16 h. MFM-300(V^IV^) retains the space group *I*4_1_22 and the overall framework structure. The V–O bond length involving the bridging oxygen reduces upon oxidation, and loss of the hydroxyl hydrogen atom in MFM-300(V^IV^) is confirmed. Analysis of the high pressure CO_2_ adsorption isotherm at 273 K by DFT/Monte-Carlo methods gives surface areas of 1892 and 1565 m^2^ g^–1^, pore size distributions (PSD) centred at 5.2 and 5.4 Å, and cumulative pore volumes of 0.490 and 0.481 cm^3^ g^–1^ for MFM-300(V^III^) and MFM-300(V^IV^), respectively. Another pair of MOFs, MIL-47(V^III^) and MIL-47(V^IV^), has structural similarity to MFM-300(V). However, MIL-47(V^III^) and MIL-47(V^IV^) show significantly different porosity owing to framework flexibility as confirmed by CO_2_ and H_2_O adsorption,^11^ and therefore cannot be directly compared in terms of host–guest binding. In comparison, MFM-300(V) adopts a rigid framework connection tied together by mutually linked *cis*-μ_2_-OH groups thus yielding highly robust isostructural frameworks for both V^III^ and V^IV^ materials.

### Analysis of adsorption and selectivity of CH_4_ and C_2_-hydrocarbons

**Fig. 2:**
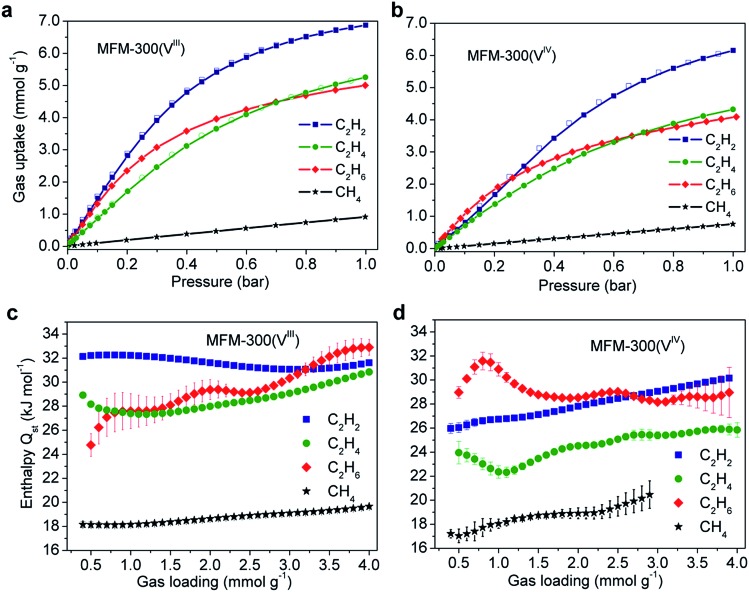
Adsorption isotherms of C_1_,_2_ hydrocarbons in (a) MFM-300(V^III^) and (b) MFM-300(V^IV^) at 303 K. Isotherms at other temperatures are shown in ESI.† Variation of isosteric heats of adsorption in (c) MFM-300(V^III^) and (d) MFM-300(V^IV^).

Gravimetric adsorption isotherms for CH_4_, C_2_H_2_, C_2_H_4_ and C_2_H_6_ have been measured for both desolvated samples at 273–303 K (Fig. 2). At 1.0 bar, the C_2_H_2_ uptake of MFM-300(V^III^) is 8.1 and 6.9 mmol g^–1^ at 273 and 303 K, respectively, and those for MFM-300(V^IV^) are 7.8 and 6.1 mmol g^–1^ under the same conditions (Fig. 2b). The density of adsorbed C_2_H_2_ in MFM-300(V^III^) is calculated to be 0.38 g cm^–3^ at 303 K, which is 184 times higher than the safe compression limit for C_2_H_2_ storage at 2.0 bar. This value is comparable to the highest value of 0.38 g cm^–3^ observed in [Zn_2_(adc)_2_(dabco)] [adc^2–^ = 9,10-anthracenedicarboxylate; dabco = 1,4-diazabicyclo[2.2.2]octane] but at a lower temperature of 296 K,^12^ and higher than those for the benchmark MOFs, such as HKUST-1 (0.31 g cm^–3^ at 296 K)^13^ and MOF-74(Fe) (0.29 g cm^–3^ at 296 K).^14^ It is interesting to note that at 273 K, the C_2_H_2_ uptake of MFM-300(V^III^) is 0.3 mmol g^–1^ higher than that of MFM-300(V^IV^) at 1.0 bar, while the difference is significantly greater (*Δ* = 1.7 mmol g^–1^) in favour of MFM-300(V^III^) at 0.1 bar (Fig. S1†). Given the similar pore volumes of these two samples, this result indicates the presence of stronger host–guest interaction in MFM-300(V^III^) than in MFM-300(V^IV^) at low surface coverage; with increasing gas loading, less surface sites are available and guest–guest interaction start to be significant in driving the gas adsorption, leading to a reduced difference in gas uptake.

The uptake capacities of C_2_ hydrocarbons of both MFM-300(V^III^) and MFM-300(V^IV^) track the order of C_2_H_2_ > C_2_H_4_ > C_2_H_6_ at 1.0 bar and 273–303 K (Fig. 2). This is reasonable considering that gas uptake is inversely correlated with the molecular size at relatively high surface coverage. Below 0.2 bar, gas uptake in MFM-300(V^III^) follows the order of C_2_H_2_ > C_2_H_6_ > C_2_H_4_, while that in MFM-300(V^IV^) follows C_2_H_6_ > C_2_H_2_ > C_2_H_4_. At low surface coverage, the effect of MOF–gas interaction is more pronounced in driving physical adsorption processes. The inversion of uptakes of C_2_H_6_ and C_2_H_2_ in MFM-300(V^III^) and MFM-300(V^IV^) indicates that the host–guest binding mechanism is very sensitive to the presence of hydroxyl groups in the pore and to the small difference in chemical structure of these hydrocarbon molecules.

**Fig. 3:**
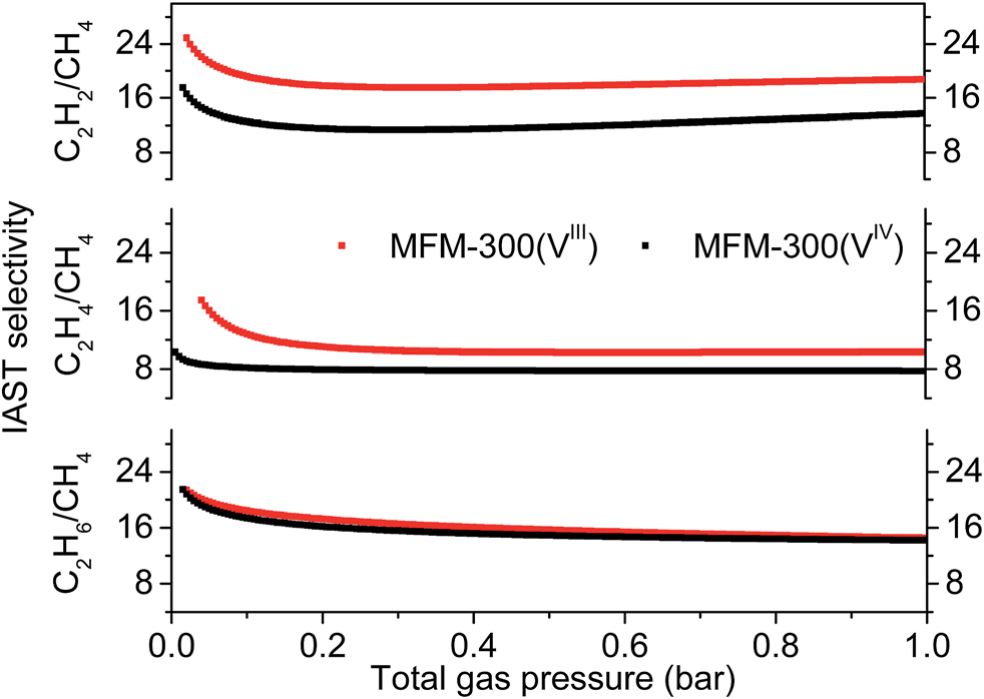
C_2_H_*n*_/CH_4_ (*n* = 2, 4, 6) IAST selectivities for MFM-300(V^III^) and MFM-300(V^IV^) calculated for equimolar mixtures at 303 K and 0–1 bar.

The IAST selectivities of equimolar mixture of C_2_H_*n*_/CH_4_ (*n* = 2, 4, 6) for MFM-300(V^III^) and MFM-300(V^IV^) were calculated at 303 K and 0–1 bar (Fig. 3). MFM-300(V^III^) outperforms MFM-300(V^IV^) for the selectivities of C_2_H_2_/CH_4_ and C_2_H_4_/CH_4_ by *ca.* 40%. This is likely due to the presence of stronger binding sites to unsaturated hydrocarbons in MFM-300(V^III^). The C_2_H_6_/CH_4_ selectivity of MFM-300(V^III^) is between 22–14, and is nearly identical to that of MFM-300(V^IV^) under same conditions. The C_2_H_6_/CH_4_ selectivity of MFM-300(V) is comparable with the benchmark MOFs, [Fe_2_(dobdc)] (20 at 313 K and 1.0 bar)^6^ and UTSA-35a (15 at 296 K and 1.0 bar).^15^ Uptake capacities are of paramount importance in determining the performance of the pressure swing adsorption process, an energetically efficient method for industrial scale separations. The uptake for C_2_H_6_ in MFM-300(V^III^) is as high as 5.0 mmol g^–1^ at 303 K and 1.0 bar, which is comparable to that of MOF-74(Fe) (5.0 mmol g^–1^ at 318 K and 1.0 bar) and much higher than that of UTSA-35a (2.4 mmol g^–1^ at 296 K and 1.0 bar), suggesting that MFM-300(V^III^) is a promising material for separation of C_2_H_6_ from CH_4_.

### Analysis of isosteric heat of adsorption

The isosteric heat of adsorption, *Q*_st_, for C_2_H_2_ in MFM-300(V^III^) is centered at 32.0 ± 0.1 kJ mol^–1^ without notable variation as a function of surface coverage. In contrast, the *Q*_st_ of C_2_H_2_ in MFM-300(V^IV^) is estimated as 26.0 ± 0.4 kJ mol^–1^ at low pressure and increases gradually with increasing gas loading to 30.1 ± 0.1 kJ mol^–1^ at 4.0 mmol g^–1^. Interestingly, the *Q*_st_ value for C_2_H_2_ in MFM-300(V^III^) is significantly higher than that of MIL-53(Al) (19.2 kJ mol^–1^), which adopts similar structural feature but with *trans*-orientated hydroxyl groups at the metal centre.^16^ Indeed, this value is even higher than that of HKUST-1 (30.4 kJ mol^–1^) incorporating open Cu^II^ center.^16^ The *Q*_st_ values of C_2_H_6_ for both compounds are similar and lie within the range of 28–32 kJ mol^–1^. The *Q*_st_ value of C_2_H_4_ in MFM-300(V^III^) (28–31 ± 0.1 kJ mol^–1^) is also higher than that of MFM-300(V^IV^) (24–26 ± 0.1 kJ mol^–1^). Finally, the *Q*_st_ values of CH_4_ are similar for both compounds and within the range of 17–19 kJ mol^–1^.

For unsaturated hydrocarbons (*i.e.,* C_2_H_2_ and C_2_H_4_), the uptake capacity and *Q*_st_ are much higher in MFM-300(V^III^) than in MFM-300(V^IV^) at low pressure, indicating the presence of possible –OH···C=C/C≡C interactions. In comparison, for saturated hydrocarbons (*i.e.*, C_2_H_6_ and CH_4_), the *Q*_st_ in both compounds are similar, suggesting the presence of similar host–guest binding mechanisms for saturated hydrocarbons in MFM-300(V^III^) and MFM-300(V^IV^). The observed undulations in the *Q*_st_ plots may suggest a packing effect reflecting optimum packing of the substrate within the pore at specific loadings.

### Determination of preferred binding sites *via* neutron powder diffraction (NPD)

In order to determine the preferred binding sites for C_2_D_2_, C_2_D_4_, C_2_D_6_ and CD_4_ in MFM-300(V), NPD experiments were carried out for gas-loaded samples of MFM-300(V^III^) and MFM-300(V^IV^) at 7 K. Comparison of the NPD patterns for bare and gas-loaded MOFs indicated that no major structural phase change took place on gas-loading, and NPD data enabled full structural analysis *via* Rietveld refinement to yield positions, orientations and occupancies of adsorbed gas molecules within the framework hosts.

**Fig. 4:**
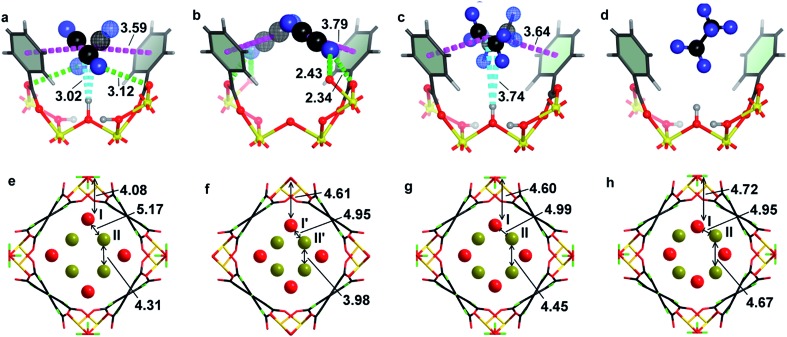
Views of the structures of gas-loaded MOFs by NPD showing the binding and packing of adsorbed hydrocarbons molecules in (a and e) MFM-300(V^III^)·1.0C_2_D_2_, (b and f) MFM-300(V^IV^)·0.7C_2_D_2_, (c and g) MFM-300(V^III^)·1.0C_2_D_4_, (d and h) MFM-300(V^III^)·1.0C_2_D_6_ (C, black; H, grey/green; D, blue; O, red, V, yellow; c.g. of adsorbed hydrocarbon molecules are shown in red and dark yellow balls).

At a loading of 1.0 C_2_D_2_/V, analysis of the NPD data revealed two crystallographically independent sites for C_2_D_2_ (I and II) (Fig. 4a and c). C_2_D_2_^I^ is in a side-on mode interacting with the hydroxyl group with a H_OH_···c.g._C≡C_ distance of 3.016(1) Å (c.g. = centre of gravity), indicating a moderate hydrogen bond between the π-electrons of C_2_D_2_ and the HO–V moiety. C_2_D_2_^II^ interacts with the carboxylate O atoms with a D···O distance of 2.608(1) and 2.871(1) Å. The H_OH_···c.g._C≡C_ distance is shorter than that found in the C_2_D_2_-loaded MFM-300(Al) [3.26(1) Å] studied by NPD at 10 K.^8^ The stronger hydrogen bond observed in MFM-300(V^III^) is likely attributed to the difference in the acidity between the Al–OH and V–OH bridges, and is consistent with the observation in the corresponding CO_2_-loaded MFM-300 systems where adsorbed CO_2_ binds to V–OH groups more strongly than to the Al–OH group.^10^,^17^ Π···π bonding is observed between the π orbitals of C_2_D_2_^I^ and the aromatic benzene rings in the carboxylate ligand, with a c.g._C≡C_···c.g._phenyl_ distance of 3.591(2) Å. Further dipole interactions are observed between adsorbed C_2_D_2_ molecules on sites I and II in a T-shape arrangement with a D···c.g._C≡C_ distance of 3.190(1) Å (Fig. S10 and S11†), comparable to that in solid C_2_H_2_ [H···c.g._C≡C_ = 3.178(1) Å].^18^ The site occupancy of adsorbed C_2_D_2_ molecules was obtained by free refinement against the NPD data. The occupancies of C_2_D_2_^I^ and C_2_D_2_^II^ are found to be 0.55 and 0.45, respectively, at a loading of 1.0 C_2_D_2_/V, indicating that site I which contacts to the hydroxyl group directly is more favourable at low pressure. As the loading increases to 1.76 C_2_D_2_/V, the occupancies of C_2_D_2_^I^ and C_2_D_2_^II^ are found to be 0.82 and 0.94, respectively, indicating the presence of intermolecular cooperative binding between adsorbed gas molecules.

Analysis of the NPD data of C_2_D_2_-loaded MFM-300(V^IV^) also shows two independent binding sites (I′, II′) for adsorbed C_2_D_2_ molecules with retention of the framework structure. C_2_D_2_^I′^ interacts with the carboxylate group with D···O_COO_ distances of 2.34(1) and 2.43(1) Å, which are slightly longer than the H_C_2_H_2__···O_COO_ hydrogen bond distance (2.2 Å) observed in [Cu_2_(pzdc)_2_(pyz)] at 170 K (pzdc^2–^ = pyrazine-2,3-dicarboxylate, pyz = pyrazine),^19^ but are shorter than the corresponding D···O_COO_ separation of 3.12(1) Å found in C_2_D_2_-loaded MFM-300(V^III^). This indicates a shift in the nature of the primary binding site on going from C_2_D_2_-loaded MFM-300(V^III^) to C_2_D_2_-loaded MFM-300(V^IV^) (Fig. 4a and b). Π···π interactions between C_2_D_2_^I′^ and the phenyl groups are also observed with a c.g._C≡C_···c.g._phenyl_ distance of 3.78(1) Å. The shortest separation between the adsorbed C_2_D_2_ molecules and the bridging oxy group is 4.83(1) Å.

Acetylene or propyne are frequently used as probes to determine the basicity of oxide materials (*e.g.*, MgO) by forming hydrogen bonds between the acidic C–H groups and the basic sites on the material surface. In this study, the absence of any notable binding interactions between adsorbed C_2_D_2_ molecules and the surface oxy groups is a direct result of the weak basicity of the oxy bridges in MFM-300(V^IV^), which, in turn, reflects the relative acidity of the hydroxyl bridges in MFM-300(V^III^), consistent with the binding observed in C_2_D_2_-loaded MFM-300(V^III^). In contrast to C_2_D_2_-loaded MFM-300(V^III^), π···π interactions are observed between the adsorbed C_2_D_2_^I′^ and C_2_D_2_^II′^ molecules in MFM-300(V^IV^) with a c.g.^I′^···c.g.^II′^ distance of 3.45(2) Å (Fig. S10 and S11†). Refinement of the site occupancies shows a 50 : 50 distribution over the two sites, and no preferred binding sites for C_2_D_2_ in MFM-300(V^IV^).

The packing of adsorbed C_2_D_2_ molecules within the two frameworks is compared in Fig. 4e and f. Overall, the host–guest interaction is stronger in MFM-300(V^III^) while the guest–guest interaction is stronger in MFM-300(V^IV^). This result suggests that the proton on the bridging oxy group can not only affect the host–guest binding through fine-turning of the pore surface chemistry, but also alter the subsequent guest–guest interaction, thus controlling the gas adsorption and substrate packing within the pores.

**Fig. 5:**
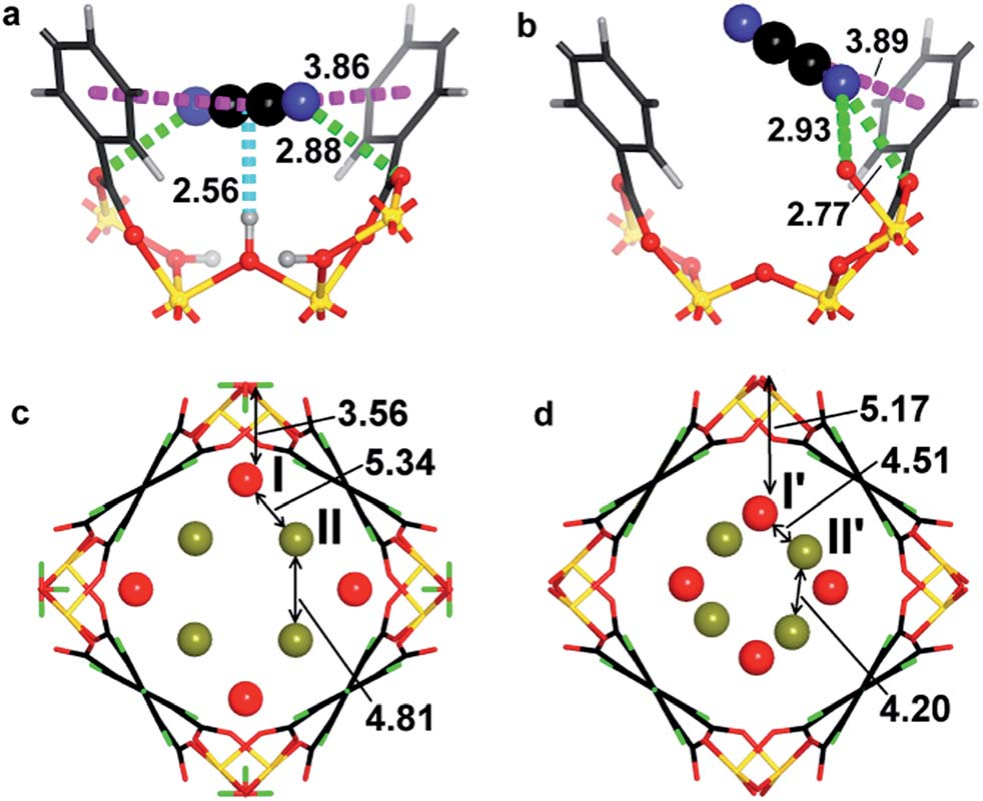
Views of the DFT-optimised structural models showing the binding and packing of adsorbed C_2_H_2_ molecules within (a and c) MFM-300(V^III^)·2.0C_2_H_2_ and (b and d) MFM-300(V^IV^)·2.0C_2_H_2_ (C, black; H, grey/green; D, blue; O, red, V, yellow; c.g. of adsorbed C_2_H_2_ molecules are shown in red and dark yellow).

The structures for C_2_D_2_-loaded MFM-300(V^III^) and MFM-300(V^IV^) have also been optimised by DFT calculations at a loading of 2.0 C_2_H_2_/V (Fig. 5). The DFT calculation for MFM-300(V^III^) confirms that the adsorbed C_2_H_2_ molecules (i) interact with the hydroxyl groups in a side-on model (H_OH_···c.g._C≡C_ = 2.56 Å); (ii) form π···π bonds with the phenyl rings of ligands (c.g._C≡C_···c.g._phenyl_ = 3.86 Å); (iii) bind with the carboxylate oxygen atom *via* dipole interactions (H_C_2_H_2__···O_carboxylate_ = 2.88 Å). In contrast, the DFT calculation for MFM-300(V^IV^) confirms (i) an absence of binding interaction between the adsorbed C_2_H_2_ molecules and the bridging oxy group; (ii) presence of π···π interaction between C_2_H_2_ molecules and the phenyl rings of ligands (c.g._C≡C_···c.g._phenyl_ = 3.89 Å); (iii) the adsorbed C_2_H_2_ molecules interact strongly with the carboxylate oxygen atom (H_C_2_H_2__···O_carboxylate_ = 2.77 Å). The shift of the binding strength for host–guest and guest–guest interaction on going from MFM-300(V^III^)·4C_2_H_2_ to MFM-300(V^IV^)·4C_2_H_2_ was clearly observed in the DFT calculation (Fig. 4c and d), and is in excellent agreement with the results from NPD experiments.

MFM-300(V^III^)·2.12C_2_H_4_ shows two binding domains for C_2_D_4_ (Fig. 3g and S12†). C_2_D_4_^I^ (occupancy = 0.56) is located close to the hydroxyl group with a c.g._C=C_···H_OH_ distance of 3.737(1) Å, which is much longer than the corresponding value of 3.016(1) Å observed in MFM-300(V^III^)·2.0C_2_D_2_. This result suggests the presence of a weaker hydrogen bond due to the reduced π-electron density in C_2_D_4_ compared to C_2_D_2_. C_2_D_4_^II^ molecules (occupancy = 0.50) contacts with the carboxylate O atoms with D···O_carboxylate_ distances in the range of 2.64(1)–3.03(2) Å. The NPD analysis of C_2_D_6_-loaded MFM-300(V^III^) suggests two sites for adsorbed C_2_D_6_ molecules, C_2_D_6_^I^ molecule is aligned at a long distance to the –OH group [distance c.g._C–C_···H_OH_ = 3.87(1) Å] as a result of no π-electron density and notable repulsion between the host and guest hydrogen atoms (Fig. 3d and h, S13†). C_2_D_6_^II^ interacts with the framework and C_2_D_6_^I^ molecule *via* van der Waals interactions. Similarly, NPD result of CD_4_-loaded MFM-300(V^III^) confirms no specific host–guest interactions but van der Waals interactions between adsorbed CD_4_ molecules and the MOF interior [distance c.g._C–C_···H_OH_ = 3.59(2) Å] (Fig. S14 and S15†).

Combining the results of NPD experiments and DFT calculations, the differences in measured *Q*_st_ between the two materials can be explained. (i) The *Q*_st_ values for C_2_H_2_ and C_2_H_4_ are higher in MFM-300(V^III^) than those in MFM-300(V^IV^) at low surface coverage, which is due to the stronger binding interaction between adsorbed C_2_H_2_ and C_2_H_4_ molecules with the framework host of MFM-300(V^III^), particularly with the free acidic hydroxyl groups *via* hydrogen bonds. (ii) The *Q*_st_ for C_2_H_2_ increases with increasing gas loading in MFM-300(V^IV^), while that for MFM-300(V^III^) remains near-constant over the entire loading, which is due to the stronger intermolecular interaction between adsorbed C_2_H_2_ molecules within the pore of MFM-300(V^IV^) as a result of lack of specific surface binding sites. (iii) The *Q*_st_ values of C_2_H_6_ and CH_4_ are similar for both MFM-300(V^III^) and MFM-300(V^IV^) because of the absence of host–guest hydrogen bonds *via* the hydroxyl groups and the van der Waals interaction drives the adsorption of C_2_H_6_ and CH_4_ in both materials.

### Inelastic neutron scattering (INS) and DFT modelling

**Fig. 6:**
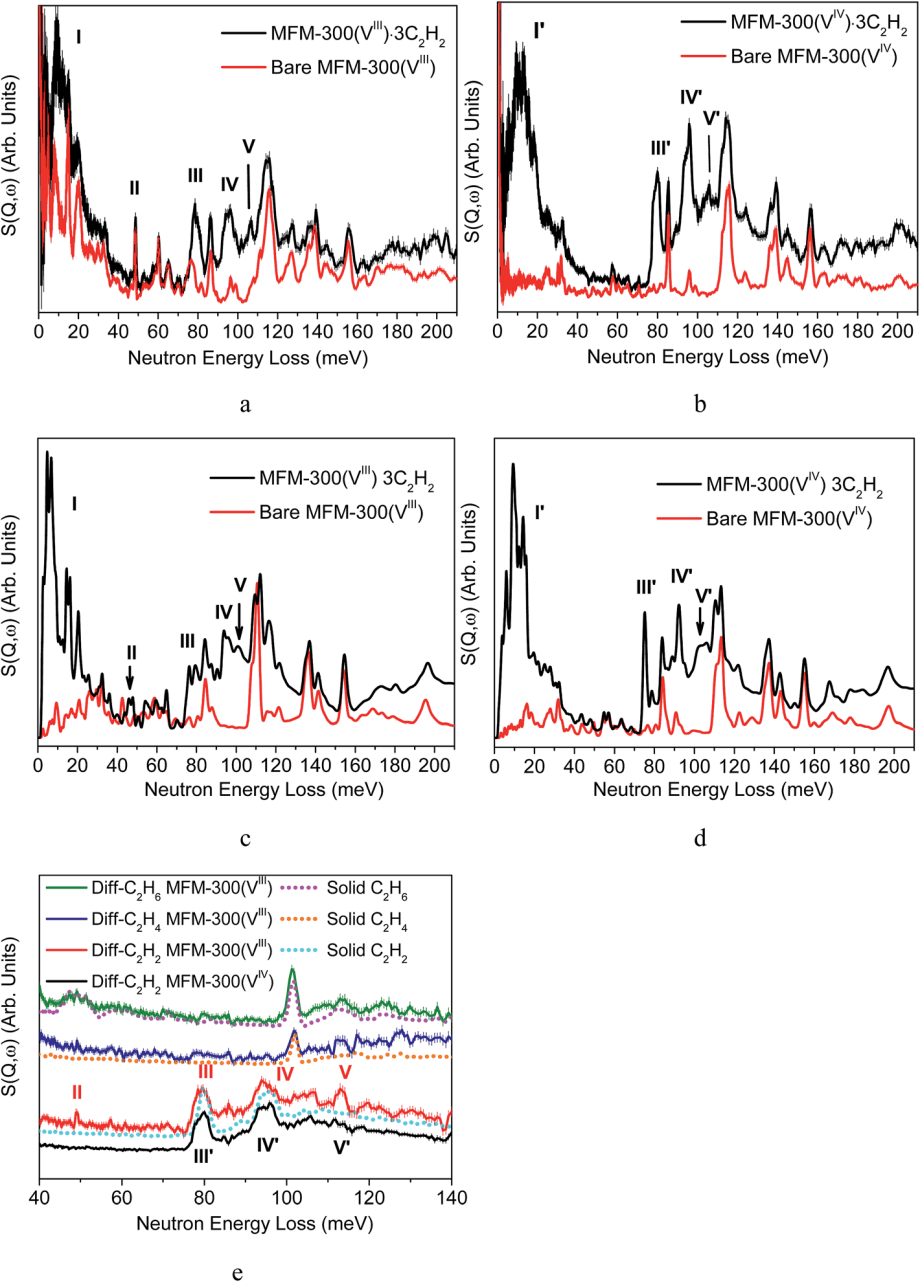
Inelastic neutron scattering (INS) spectra for gas-loaded MFM-300(V). Experimental INS spectra for (a) MFM-300(V^III^)·3C_2_H_2_ and (b) MFM-300(V^IV^)·3C_2_H_2_. DFT simulated INS spectra for (c) MFM-300(V^III^)·4C_2_H_2_ and (d) MFM-300(V^IV^)·4C_2_H_2_. (e) Difference spectra for gas-loaded MFM-300(V) and INS spectra for solid C_2_ hydrocarbons.

In addition to static crystallographic studies, we have combined INS and DFT to directly visualise the binding dynamics of adsorbed C_2_H_2_, C_2_H_4_, C_2_H_6_ and CH_4_ molecules in MFM-300(V^III^) and C_2_H_2_ in MFM-300(V^IV^) (Fig. 6). INS is a powerful neutron spectroscopic technique which is particularly sensitive to the dynamics of hydrogen atoms owing to the exceptionally large cross section for that isotope. The INS spectra of condensed C_2_H_2_, C_2_H_4_ and C_2_H_6_ in the solid state were also collected for comparison. The INS spectrum for bare MFM-300(V^III^) shows a series of peaks in the range of 40–60 MeV attributed to the deformational modes of the hydroxyl group, and the disappearance of these peaks in the INS spectrum of MFM-300(V^IV^) unambiguously confirms the deprotonation of the bridging hydroxyl group upon oxidation.

Comparison of INS spectra, measured at temperatures below 11(±0.1) K to minimize the thermal motion of the adsorbed hydrocarbons and the host, reveals five major changes in peak intensity on going from bare MFM-300(V^III^) to MFM-300(V^III^)·3C_2_H_2_ (Fig. 6a). Peaks I and II occur at a low energy transfer (5–24 and 25–50 MeV, respectively) and peaks III, IV and V at high energy transfer (79, 94 and 113 MeV, respectively). The difference spectra were obtained by subtracting the spectrum of bare MOF from that of gas-loaded MOFs, and are shown in Fig. 6e together with the spectrum of condensed C_2_H_2_ in solid state. To understand these changes, DFT calculations were used to simulate the INS spectra and optimise the structures of bare and C_2_H_2_-loaded MFM-300(V^III^). On convergence, excellent agreement between the calculated and experimental structural models were obtained (Fig. 6c and d). Peaks I, III and IV can be assigned to translational, asymmetric and symmetric C–H vibrational motions of adsorbed C_2_H_2_ molecules, respectively.^8^ Peak II can be assigned to the –OH groups bending/wagging perpendicular to the V^III^–O–V^III^ plane and peak V to the bending and out-of-plane wagging of the aromatic C–H groups on two benzene rings (Fig. 6e). The significant changes observed for peaks II suggest that adsorbed C_2_H_2_ molecules have a direct interaction with the –OH groups, and thereby affect their molecular motions and induce the changes in the INS spectra.

In contrast, addition of C_2_H_2_ in MFM-300(V^IV^) is accompanied by the appearance of four new peaks in the INS spectrum (Fig. 6b). Peaks I′, III′, and IV′ can be assigned to translational, asymmetric and symmetric C–H vibrational motions of adsorbed C_2_H_2_ molecules, respectively, and peak V′ to the bending and out-of-plane wagging of the aromatic C–H groups on the benzene rings, which are similar to the observation of MFM-300(V^III^). No major changes in the intensity at 40–70 MeV, which is in a sharp contrast to that in the INS study of MFM-300(V^III^) (Fig. 6e).

In the case of C_2_H_4_-loaded MFM-300(V^III^), small changes in intensity of peaks at 47 and 115 MeV were observed upon addition of C_2_H_4_, which are attributed to the interaction of adsorbed C_2_H_4_ with the hydroxyl and C–H groups from phenyl rings, respectively (Fig. 6e and S17†). From the difference INS plot of C_2_H_6_-loaded MFM-300(V^III^), it is clear that all peaks are ascribed to the adsorbed C_2_H_6_ molecules, and no specific host–guest interaction was seen (Fig. 6e and S18†). Similarly, addition of CH_4_ in MFM-300(V^III^) does not induce an obvious change in the INS spectra either (Fig. S19†). These results confirm near solid-type motion of adsorbed C_2_H_6_ and CH_4_ gases within the pore of MFM-300(V^III^), in excellent agreement with the NPD study and the *Q*_st_ analysis.

Thus, the INS and DFT results confirm that (i) in MFM-300(V^III^), both –OH and –CH/phenyl ring are active binding sites for adsorbed C_2_H_2_ molecules; (ii) in MFM-300(V^IV^), –CH/phenyl ring is the sole binding sites for adsorbed C_2_H_2_ molecules *via* formation of supramolecular contacts. These observations are entirely consistent with the NPD studies and confirm the significant effects of bridging hydroxyl groups on the guest binding in the host on a molecular level.

## Conclusions

We report the adsorption of light hydrocarbons in a pair of iso-structural MOFs, MFM-300(V^III/IV^), incorporating redox-active vanadium centers. The oxidation of V centers from III to IV induces deprotonation of the bridging hydroxyl group, achieving fine-tuning of the pore environment. It has been confirmed in this study that these protons play a key role in adsorption of unsaturated hydrocarbons (both uptakes and isosteric heats of adsorption). A comprehensive study combining NPD, INS and DFT modelling has unambiguously determined the preferred binding sites and structural dynamics for these host–guest systems. The differences in C_2_H_*n*_/CH_4_ (*n* = 2, 4, 6) selectivities between MFM-300(V^III^) and MFM-300(V^IV^) are fully rationalised. The acidic bridging hydroxyl groups in the pore provides specific binding activity to unsaturated hydrocarbons *via* formation of hydrogen bonds, leading to both high selectivity and packing efficiency of adsorbed gas molecules.

## Conflicts of interest

The authors declare no competing financial interests.

## Supplementary Material

Crystal structure data

Supplementary information

Crystal structure data

Crystal structure data

Crystal structure data

Crystal structure data

Crystal structure data

Crystal structure data

Crystal structure data

Crystal structure data
