# Supplementary material for: Direct observation of supramolecular binding of light hydrocarbons in vanadium(iii) and (iv) metal–organic framework materials
Source: Chem Sci. 2018 Feb 21;9(13):3401–8. doi: 10.1039/c8sc00330k (PMC5933292; doi:10.1039/c8sc00330k)
Supplement: Supplementary file 2 [file SC-009-C8SC00330K-s002.pdf]

Electronic Supplementary Material (ESI) for Chemical Science.
This journal is © The Royal Society of Chemistry 2018

Supplementary Information

Direct observation of supramolecular binding of light hydrocarbons in vanadium (III) and (IV) metal-organic framework materials

Zhenzhong Lu,[1,2] Harry G.W. Godfrey,[1] Ivan da Silva,[3] Yongqiang Cheng,[4] Mathew Savage,[1] Pascal Manuel,[3] Svemir Rudić,[3] Anibal J. Ramirez-Cuesta,[4] Sihai Yang[1]* and Martin Schröder[1]*

[1] School of Chemistry, University of Manchester, Oxford Road, Manchester, M13 9PL (UK)

Sihai.Yang@manchester.ac.uk; M.Schroder@manchester.ac.uk

[2] Institute of Advanced Materials (IAM), Nanjing Tech University, Nanjing, 210009 (P. R. China)

[3] ISIS Facility, STFC Rutherford Appleton Laboratory, Chilton, Oxfordshire, OX11 0QX (UK)

[4] The Chemical and Engineering Materials Division (CEMD), Neutron Sciences Directorate, Oak Ridge National Laboratory, Oak Ridge, TN 37831 (USA)

Experimental

Materials and measurements.

All reagents were used as received from commercial suppliers without further purification. Analyses for C, H and N were carried out on a CE-440 elemental analyzer (EAI Company). Powder X-ray diffraction data (PXRD) were collected over the 2θ range 4-50° on a Bruker Advance D8 diffractometer using Cu-Kα_1 radiation (λ = 1.54056 Å, 40 kV/40mA).

Synthesis of single crystals of MFM-300(V^{III})-solv

A mixture of VCl_3 (0.076 g, 0.48 mmol), deionised water (5.0 mL) (degassed previously under Ar for 0.5 h), biphenyl-3,3',5,5'-tetracarboxylic acid (0.010 g, 0.03 mmol) and hydrochloric acid (1.6 M, 1.0 mL) was transferred into a 23mL Teflon lined autoclave which was then charged with argon and sealed. The autoclave was heated at 210 ^{o}C for 3 days to afford dark green prism-shaped crystals accompanied with colourless block-shaped crystals; these are MFM-300(V^{III})-solv and the re-crystallised ligand, respectively. The product was soaked in fresh *N,N'*-dimethylformamide (DMF) overnight to remove any re-crystallised or free ligand, and crystals of pure MFM-300(V^{III})-solv (ca. 5% yield) were collected by filtration and preserved in acetone.

Synthesis of bulk MFM-300(V^{III})-solv

A mixture of VCl_3 (0.40g, 2.55 mmol) in deionised water (10.0mL) (degassed previously under Ar for 0.5 h), biphenyl-3,3',5,5'-tetracarboxylic acid (0.14g, 0.42 mmol), hydrochloric acid (0.3 M. 2.0mL) was transferred in to a 45mL Teflon lined autoclave which was then charged with Ar and sealed. The reaction was heated at 210 ^{o}C for 3 days to produce MFM-300(V^{III})-solv as a green microcrystalline powder (90% yield). This was soaked in fresh DMF overnight, and collected by filtration and stored under acetone.

Preparation of desolvated MFM-300(V^{III})

MFM-300(V^{III})-solv was placed in Schlenk flask, which was evacuated to $<1\times10^{-3}$ mbar at ambient temperature for 1 h, and then heated at 150 ^{o}C for overnight under vacuum. Activated MFM-300(V^{III}) was

obtained as a green powder and stored in glovebox under N_2. An important issue in desolvating MFM-300(V^{III}) is to avoid oxidation. Elemental analysis for MFM-300(V^{III}), $C_{16}H_8O_{10}V_2$ [found (calculated%)]: C 41.4(41.6), H 1.9(1.7), N 0.0(0.0).

Post-synthetic oxidation of MFM-300(V^{III}) to MFM-300(V^{IV})

Single crystals or powder MFM-300(V^{III}) was heated under pure O_2 flow at 150 °C overnight using a tube furnace. The temperature ramping speed was set to 1.0 °C/minute. MFM-300(V^{IV}) was obtained as dark purple solid which was stored under N_2 in a glovebox. Elemental analysis for MFM-300(V^{IV}), $C_{16}H_6O_{10}V_2$ [found (calculated)%]: C 41.5(41.8), H 1.5(1.3), N 0.0(0.0).

Hydrocarbon adsorption

CH_4, C_2H_2, C_2H_4 and C_2H_6 sorption isotherms were recorded at 273–303 K on an IGA-003 system under ultrahigh vacuum. The CH_4, C_2H_4 and C_2H_6 gases used were of ultrapure research grade (99.99%). C_2H_2 was purified by dual-stage cold-trap systems operated at 195 K (dry ice) before introduction to the IGA system.

Neutron Powder Diffraction (NPD) Experiments and Structure Determination.

Neutron powder diffraction experiments were carried out at WISH, a long wavelength powder and single crystal neutron diffractometer at the ISIS Facility at the Rutherford Appleton Laboratory (UK). The instrument views a solid methane moderator providing a high flux of cold neutrons with a large bandwidth, transported to the sample *via* an elliptical guide. The WISH divergent jaws system allows tuning the resolution according to the need of the experiment; in this case, it was set up in high resolution mode. The WISH detectors are 1m long, 8mm diameter pixellated 3He tubes positioned at 2.2m from the sample and arranged on a cylindrical locus covering 10-170 degrees in 2θ scattering angle. To reduce the background from sample environment, it is equipped with an oscillating radial collimator that defines a cylinder of radius about 22 mm at 90 degrees scattering.

A sample of desolvated MFM-300(V^{III}) was loaded into a cylindrical vanadium sample container with an

indium vacuum seal and connected to a gas handling system. The sample was degassed at 10^{-7} mbar and 100 °C for 1 day to remove any remaining trace guest water molecules. The temperature during data collection was controlled using a helium cryostat (7 ± 0.2 K). The loadings of hydrocarbons (for example, described for C_2D_2 hereafter) were performed by the volumetric method at ambient temperature to ensure that C_2H_2 was present in the gas phase when not adsorbed and also to ensure sufficient mobility of C_2D_2 inside the crystalline structure of MFM-300(V^{III}). NPD data were collected for the bare material, and the material dosed with 1.0 and 2.0 C_2D_2 per vanadium. The sample was then slowly cooled down to 7 K to ensure C_2D_2 was completely adsorbed with no condensation in the cell. Sufficient time was allowed to achieve thermal equilibrium before data collection. Rietveld refinements on the NPD patterns of the bare MOF and the samples at various C_2D_2 loadings were performed using the TOPAS software package. The initial Fourier difference maps were used to find the isosurfaces of the three-dimensional difference scattering-length density distribution for C_2D_2 molecules. In this treatment the C_2D_2 molecules were treated as rigid bodies; we first refined the centers of mass, orientations, and occupancies of the adsorbed C_2D_2, followed by full profile Rietveld refinement including the positions of metals and linkers, together with their corresponding lattice parameters, resulting in satisfactory R-factors. The final refinements on all the parameters including fractional coordinates, thermal parameters, occupancies for both host lattice and adsorbed C_2D_2 molecules, and background/profile coefficients yielded very good agreement factors. No restriction of the molecule position was used in the refinement. The total occupancies of C_2D_2 molecules obtained from the refinement are also in good agreement with the experimental values for the C_2D_2 loading.

Inelastic Neutron Scattering (INS)

INS experiments were undertaken using the TOSCA spectrometer at the ISIS facility. MFM-300(V) was loaded into an 11 mm diameter vanadium sample can and outgassed at 1 x 10^{-7} mbar and 100 °C for 1 day. The sample was loaded into a helium closed cycle refrigerator (CCR) cryostat and cooled to 11 K for data

collection. The hydrocarbon gas was introduced by warming the samples to 290 K, and the gas dosed volumetrically from a calibrated volume. The gas-loaded sample was then cooled to 11 K over a period of 2 h to ensure good mobility of adsorbed hydrocarbon within the crystalline structure of MFM-300(V). The sample was kept at 11 K for an additional 30 mins before date collection to ensure the thermal equilibrium (see Supplementary method for more details of DFT Calculation for INS spectra)

DFT Calculation for INS spectra

Modelling by Density Functional Theory (DFT) of the bare and C_2H_2-loaded MOFs was performed using the Vienna Ab initio Simulation Package (VASP).[1] The calculation used Projector Augmented Wave (PAW) method[2,3] to describe the effects of core electrons, and Perdew-Burke-Ernzerhof (PBE)[4] implementation of the Generalized Gradient Approximation (GGA) for the exchange-correlation functional. Energy cutoff was 900eV for the plane-wave basis of the valence electrons. The lattice parameters and atomic coordinates determined by NPD in this work were used as the initial structure. Some of the C_2H_2 sites have partial occupancy, and to account for this properly a supercell calculation would be desirable, but too costly in practice. Instead, a single unit cell was used and the partially occupied sites were modified to be either occupied or unoccupied, according to their local environment and symmetry (there needs to be either a complete C_2H_2 molecule or no molecule, and the overall probability of being occupied needs to be proportional to the actual occupancy). The total energy tolerance for electronic energy minimization was 10^{-8} eV, and for structure optimization it is 10^{-7} eV. The maximum interatomic force after relaxation was below 0.005 eV/Å. The optB86b-vdW functional[5] for dispersion corrections was applied. The vibrational eigen-frequencies and modes were then calculated by solving the force constants and dynamical matrix using Phonopy.[6] The O'Climax software[7] was used to convert the DFT-calculated phonon results to the simulated INS spectra.

The structures relaxed by the DFT calculation slightly deviate from the ones obtained from NPD experiments (*e.g.*, the orientation of the C_2H_2 molecule in Fig. 5a). This is not unusual, and in this case, the

main reason could be associated with how “partial occupancy” is handled in the simulation, as described above. To be more specific, DFT phonon calculations are usually computationally intensive and one cannot afford very large supercells with no symmetry. The above approach was used in order to best capture the local structure while still having the simulation feasible. But, the "randomness" associated with the partial occupancy cannot be reproduced fully, and the periodic boundary conditions inevitably results in certain symmetry constraints that are not present in the real material or the NPD data refinement. These discrepancies can lead to the different orientation of the C_2H_2 molecules, and it appears to be more prominent in the case of MFM-300(VIII). It should be noted that other factors may also play a role, such as insufficient treatment of the van-der-Waals interactions in DFT.

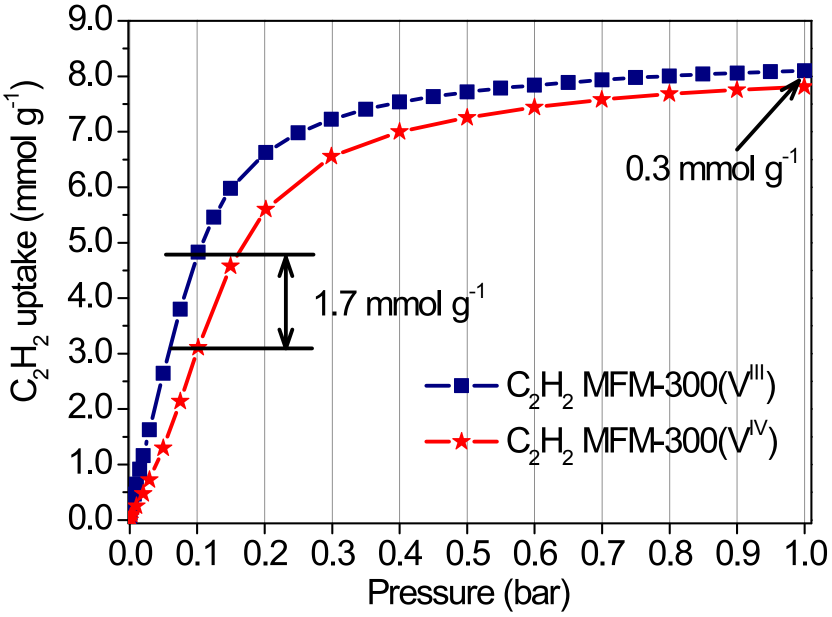

Fig. S1: Adsorption isotherms for C_2H_2 in MFM-300(V^{III}) and MFM-300(V^{IV}) showing the uptake differences at low and higher pressures at 273K.

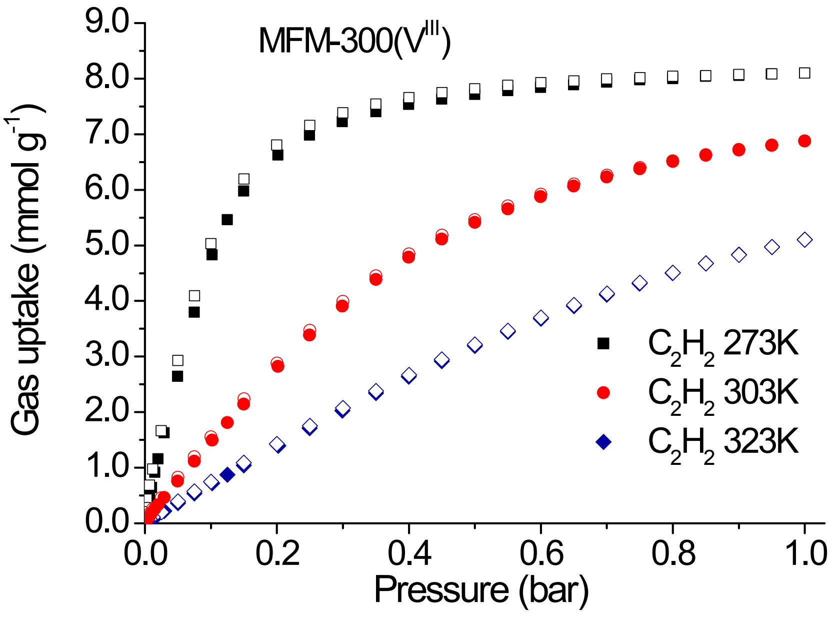

Fig. S2: Adsorption isotherms for C_2H_2 in MFM-300(V^{III}) at 273, 303 and 323 K.

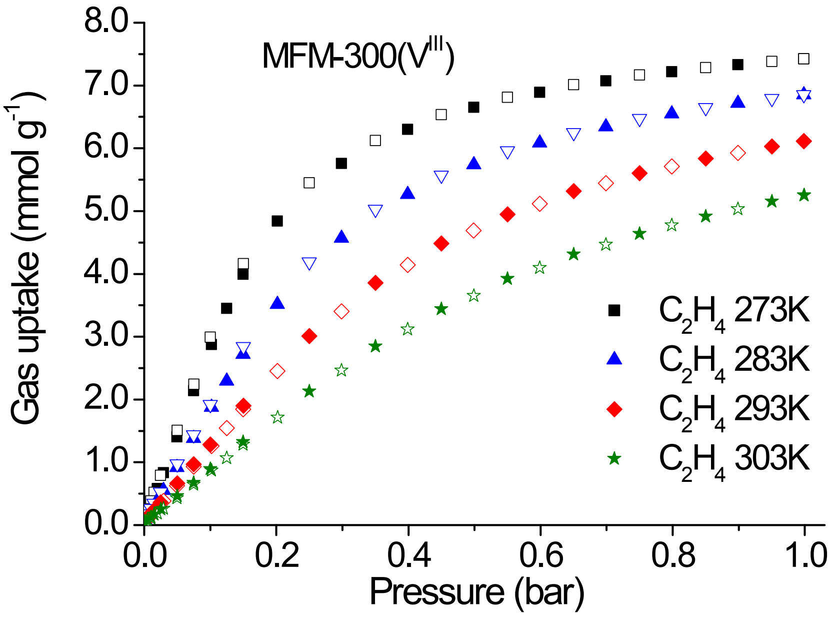

Fig. S3: Adsorption isotherms for C_2H_4 in MFM-300(V^{III}).

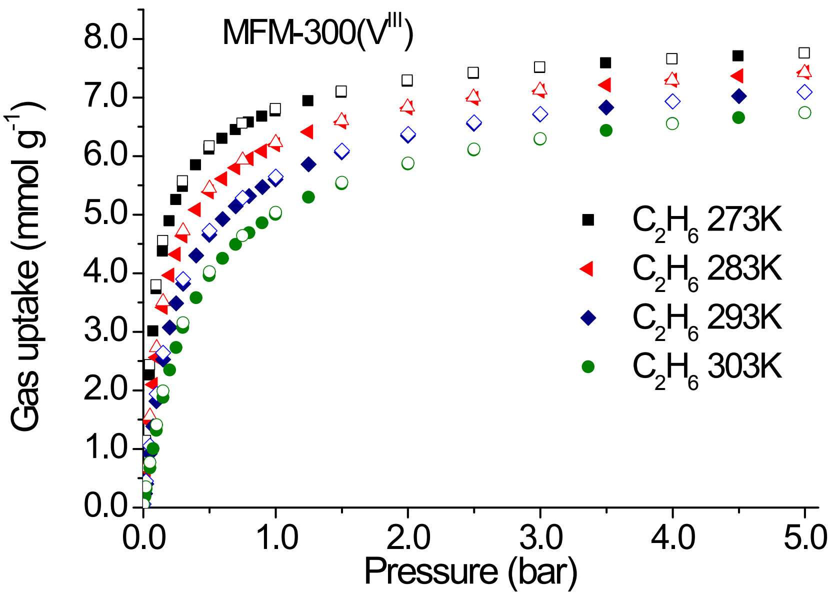

Fig. S4: Adsorption isotherms for C_2H_6 in MFM-300(V^{III}).

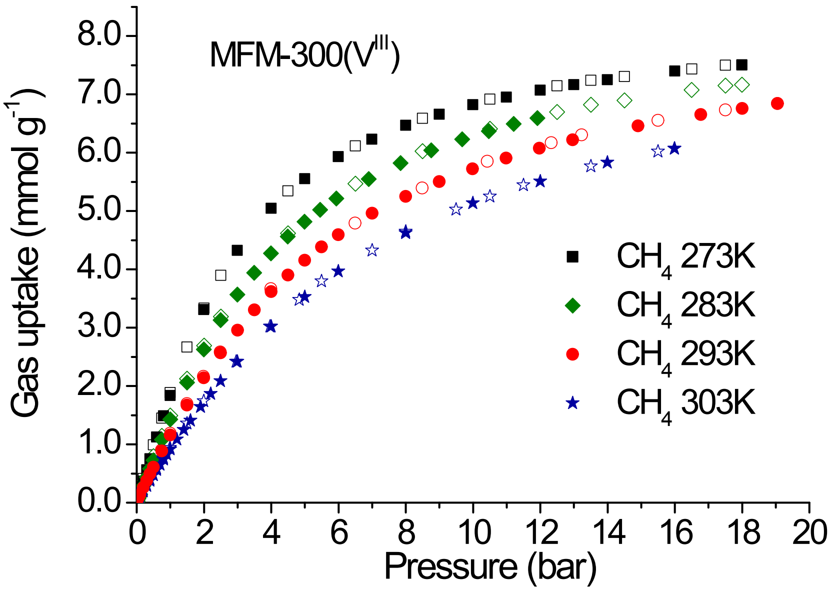

Fig. S5: Adsorption isotherms for CH_4 in MFM-300(V^{III}).

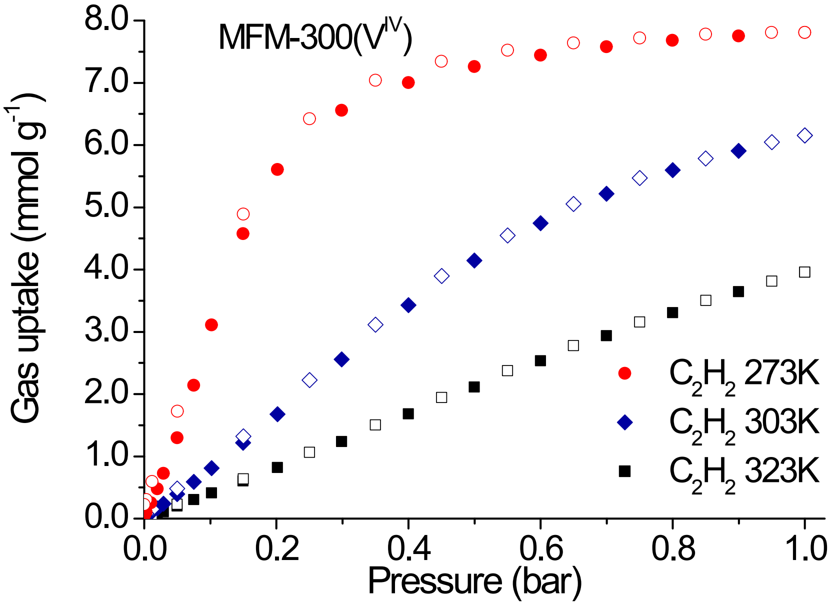

Fig. S6: Adsorption isotherms for C_2H_2 in MFM-300(V^{IV}).

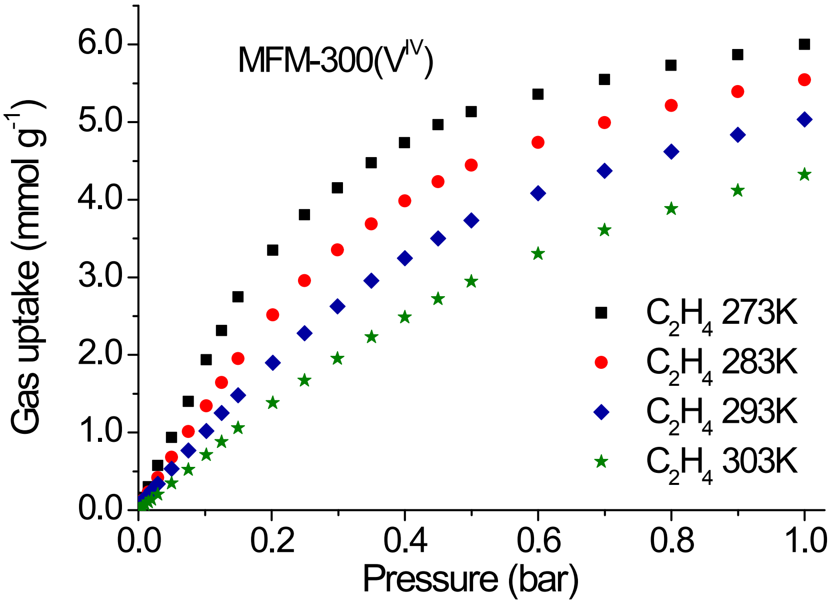

Fig. S7: Adsorption isotherms for C_2H_4 in MFM-300(V^{IV}).

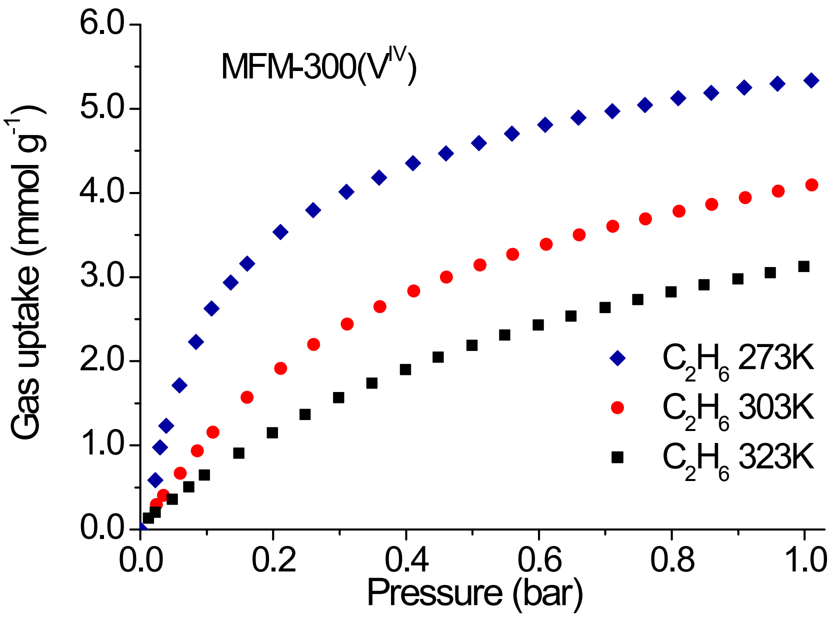

Fig. S8: Adsorption isotherms for C_2H_6 in MFM-300(V^{IV}).

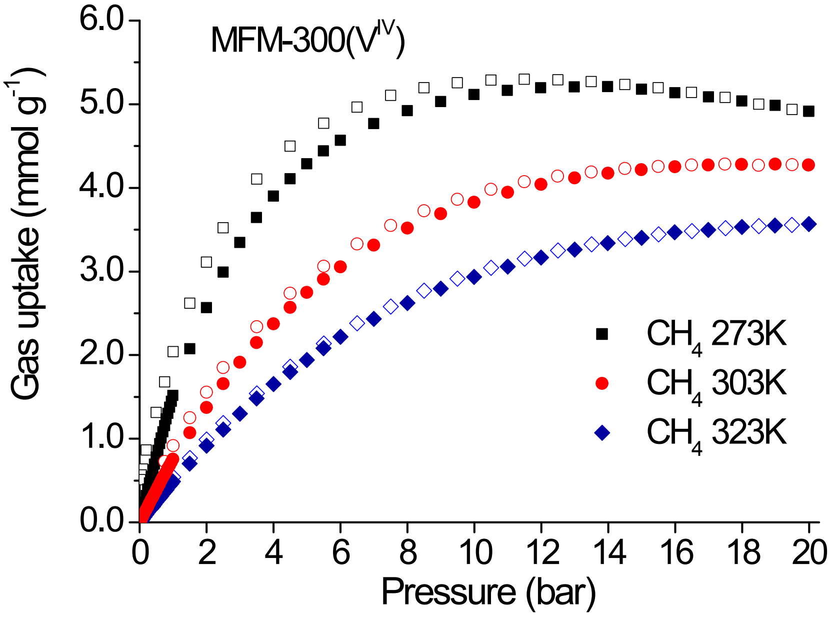

Fig. S9: Adsorption isotherms for CH_4 in MFM-300(V^{IV}). The small decrease of uptake at 273 K in the isotherm at high pressure are subject to uncertainties of the instrument because the sample has reached adsorption saturation at *ca*. 12 bar.

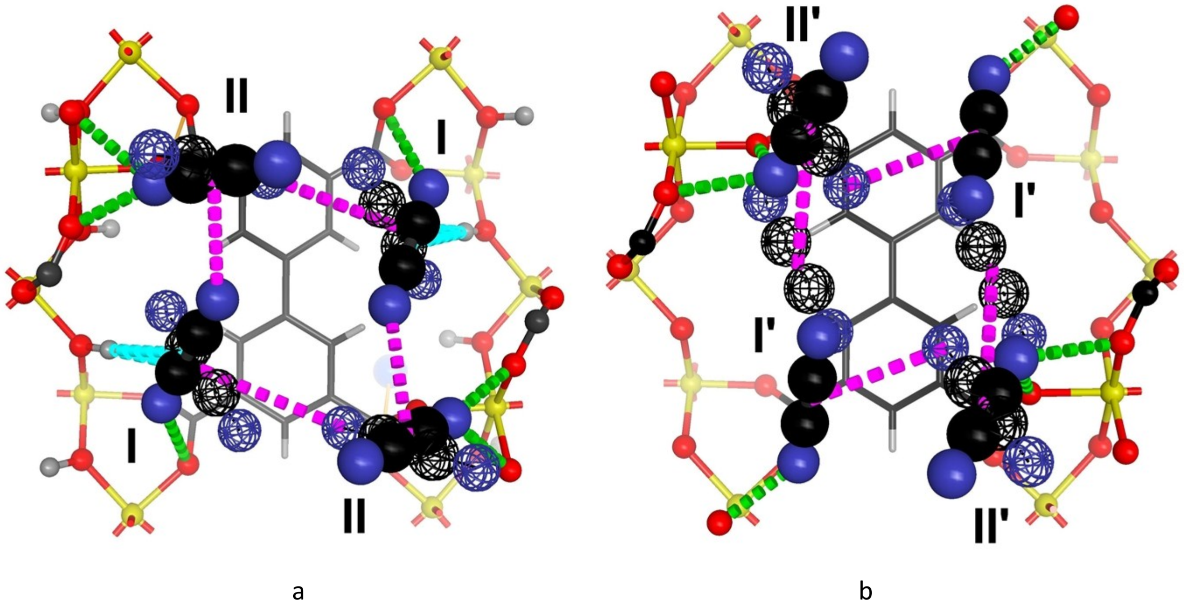

Fig. S10. Views of adsorbed C_2D_2 molecules at sites I(I') and II(II') determined by NPD in (a) MFM-300(V^{III}) and (b) MFM-300(V^{IV}). The adsorbed C_2D_2 molecules at both site I(I') and II(II') are disordered over two positions; one of the disordered parts is shown in hollow ball mode.

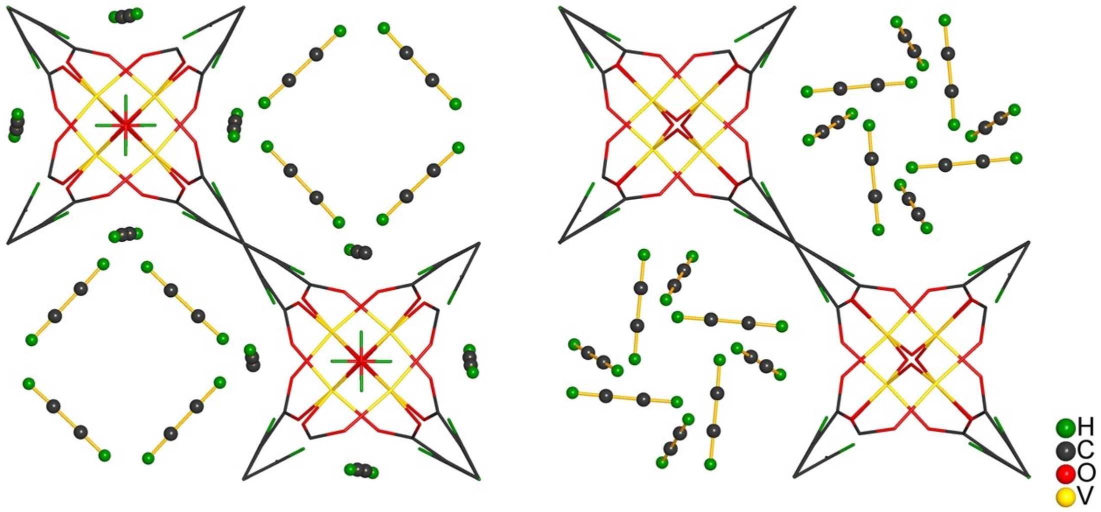

a b

Fig. S11. Views of positions of adsorbed C_2H_2 molecules at sites I(I') and II(II') in (a) MFM-300(V^{III}) and (b) MFM-300(V^{IV}) determined by DFT.

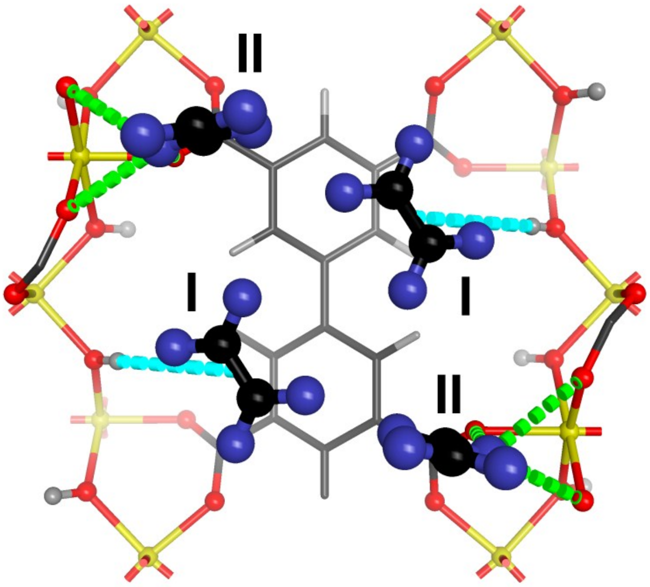

Fig. S12: Views of C_2D_4 molecules at sites I and II in MFM-300(V^{III}).

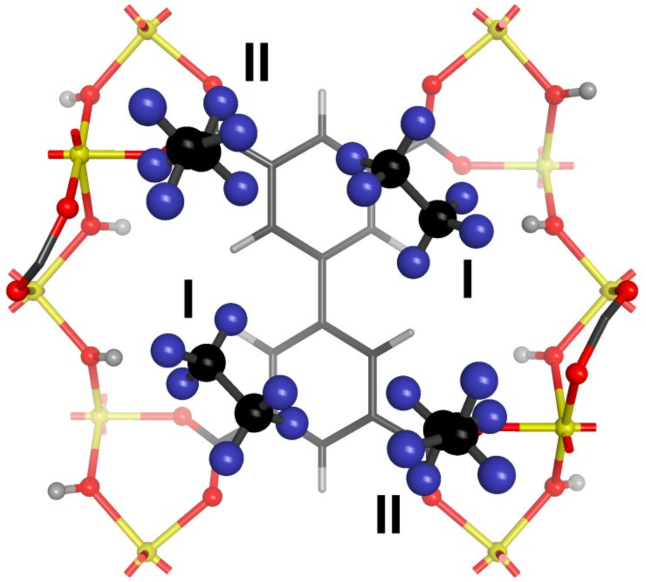

Fig. S13: Views of C_2D_6 molecules at sites I and II in MFM-300(V^{III}).

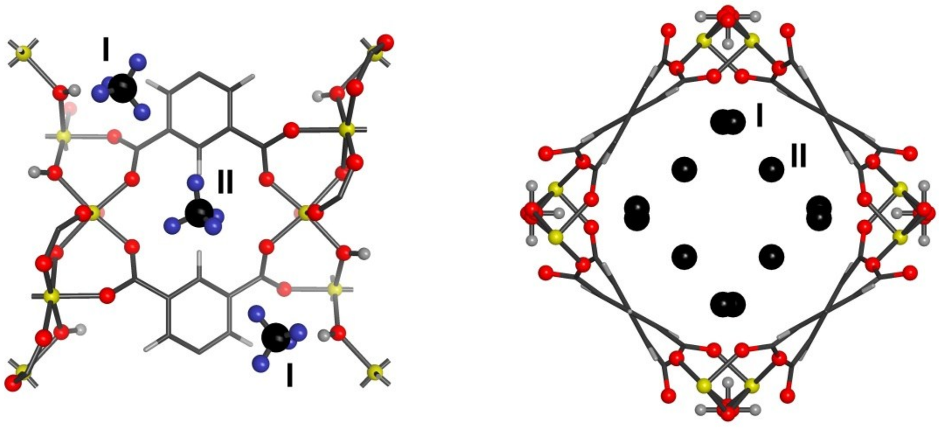

Fig. S14: Views of CD_4 molecules at sites I and II (left) and packing of the centres of the adsorbed CD_4 (right) in MFM-300(V^{III})·1.0CD_4.

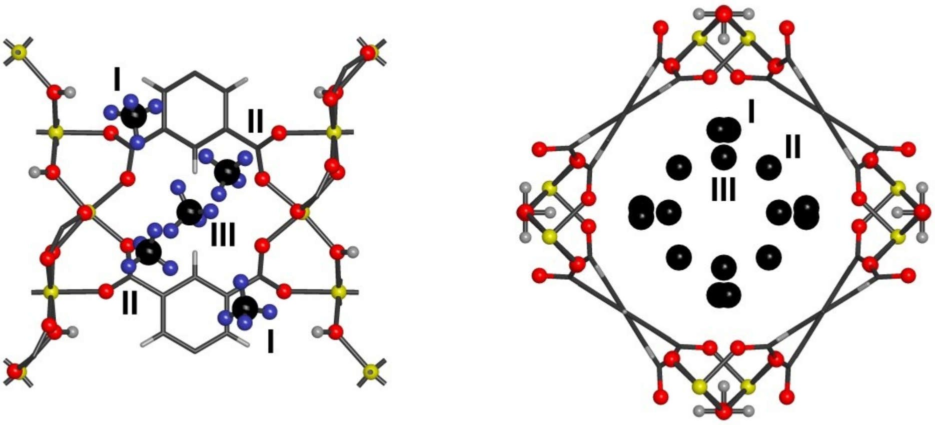

Fig. S15: Views of CD_4 molecules at sites I, II and III (left) and packing of the centres of the adsorbed CD_4 (right) within MFM-300(V^{III})·1.4CD_4.

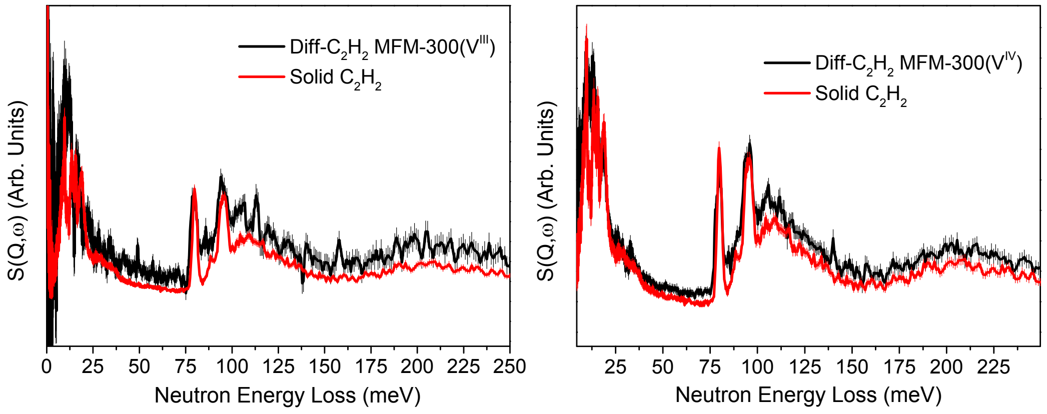

Fig. S16: Difference INS spectra for C_2H_2-loaded MFM-300(V^{III}) (left) and MFM-300(V^{IV}) (right) and INS spectra for solid C_2H_2.

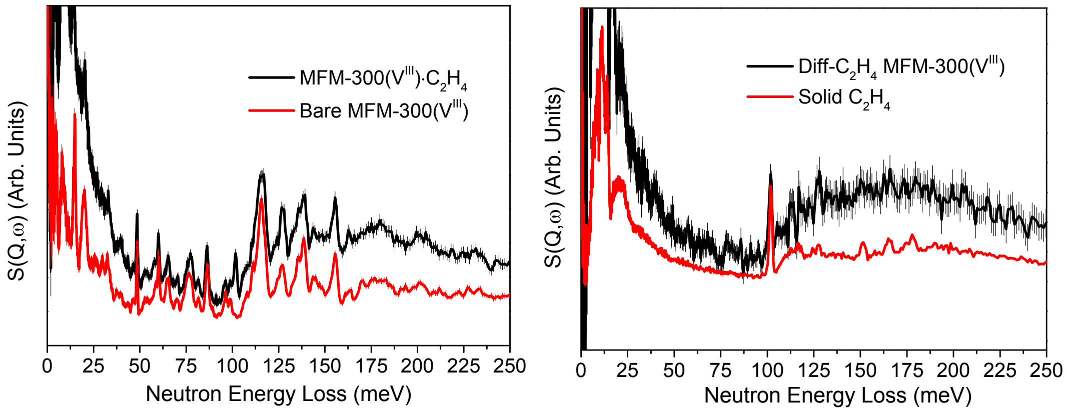

Fig. S17: Left, experimental INS spectra for bare and C_2H_4-loaded MFM-300(V^{III}). Right, difference INS spectra for MFM-300(V^{III})·C_2H_4 and INS spectra for solid C_2H_4.

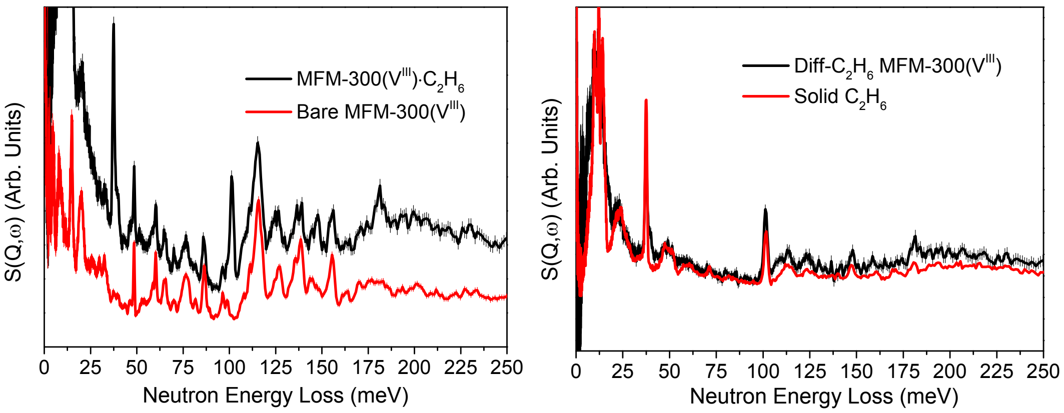

Fig. S18: Left, experimental INS spectra for bare and C_2H_6-loaded MFM-300(V^{III}). Right, difference INS spectra for MFM-300(V^{III})$\cdot C_2H_6$ and INS spectra for solid C_2H_6.

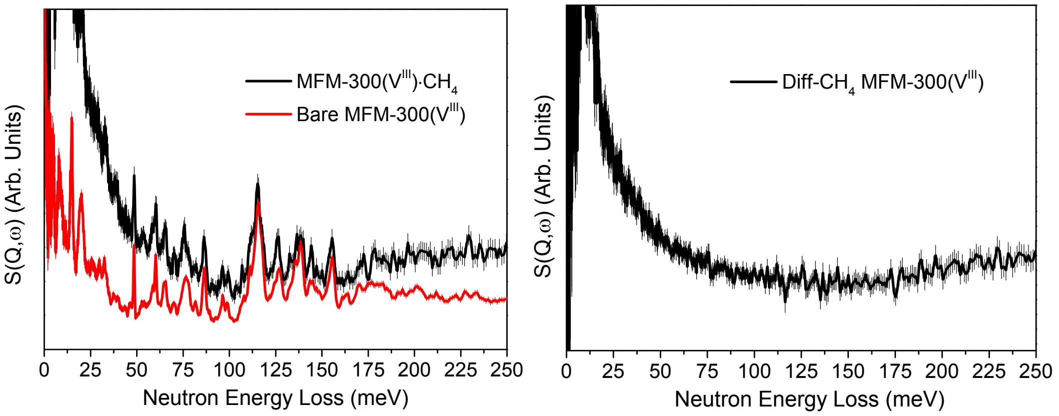

Fig. S19: Left, experimental INS spectra for bare and CH_4-loaded MFM-300(V^{III}). Right, difference INS spectra for MFM-300(V^{III})$\cdot CH_4$.

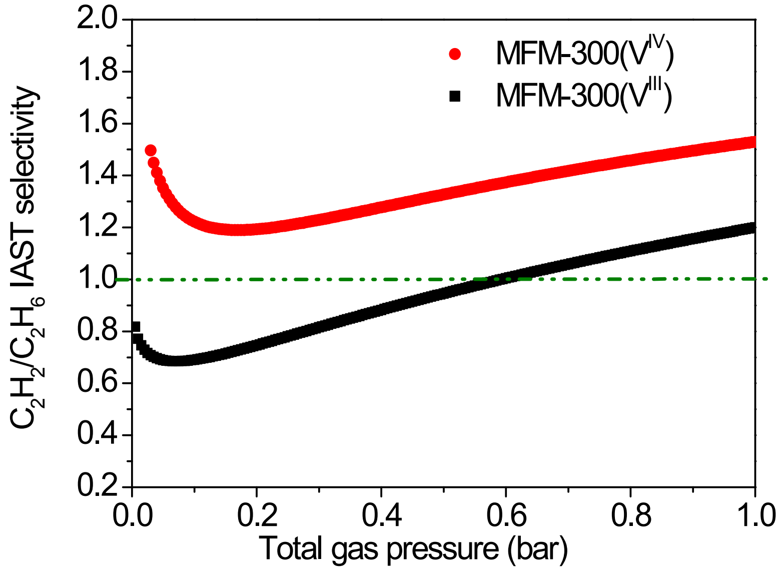

Fig. S20: C_2H_2/C_2H_6 IAST selectivities for MFM-300(V^{III}) and MFM-300(V^{IV}) calculated at equimolar mixture at 303K.

References

1. Kresse, G. & Furthmüller, J. Efficient iterative schemes for ab initio total-energy calculations using a plane-wave basis set. *Phys. Rev. B*, **54**, 11169-11186 (1996).
2. Blochl, P. E. Projector augmented-wave method. *Phys. Rev. B*, **50**, 17953-17979 (1994).
3. Kresse, G. & Joubert, D.From ultrasoft pseudopotentials to the projector augmented-wave method. *Phys. Rev. B*, **59**, 1758-1775 (1999).
4. Perdew, J. P., Burke, K. & Ernzerhof, M. Generalized gradient approximation made simple. *Phys. Rev. Lett.*, **77**, 3865-3868 (1996).
5. Klimeš, J., Bowler, D. R. & Michaelides, A. Chemical accuracy for the van der Waals density functional. *J. Phys.: Cond. Matt.* **22**, 022201 (2010).
6. Togo, A. & Tanaka, I. First principles phonon calculations in materials science. *Scr. Mater.*, **108**, 1-5 (2015)
7. Ramirez-Cuesta, A. J. O'Climax 4.0.1, The new version of the software for analysing and interpreting INS spectra. *Comput. Phys. Comm.***157**, 226-238 (2004).
